# Authors’ Reply to: Methodological Clarifications and Generalizing From Weibo Data. Comment on “Nature and Diffusion of COVID-19–related Oral Health Information on Chinese Social Media: Analysis of Tweets on Weibo”

**DOI:** 10.2196/29145

**Published:** 2021-05-21

**Authors:** Zhuo-Ying Tao, Yu-Xiong Su

**Affiliations:** 1 Division of Oral and Maxillofacial Surgery Faculty of Dentistry The University of Hong Kong Hong Kong Hong Kong (China)

**Keywords:** COVID-19, dentistry, oral health, dental health, online health, social media, tweet, Weibo, China, health information

We sincerely thank Yadav [1] for the comments on our paper, “Nature and Diffusion of COVID-19–related Oral Health Information on Chinese Social Media: Analysis of Tweets on Weibo” [2]. We appreciate the insightful and constructive opinions on this topic. Indeed, oral health information on social media during the pandemic is important and worthy of in-depth study. Here, we would like to further discuss this topic.

The aim of our study was to investigate oral health information on Chinese social media during the COVID-19 outbreak. As a scientific publication with open access, this study is available to any member of the general public who is interested in this topic; thus, our paper is not oriented toward a specific audience. In this study, the nature and diffusion of COVID-19–related oral health information on Weibo was overviewed. The government can get useful ideas for public health surveillance or policymaking during the epidemic, while dental care providers and potential patients might be inspired knowing that social media is a good communication tool for exchanging oral health knowledge and dental care needs during an emerging crisis.

In terms of the keywords used in this study, we only selected “stomatology” and “dentistry” because these 2 keywords in Chinese provide broad coverage, including oral, mouth, tooth, etc. As such, when we conducted the search with “stomatology” and “dentistry,” tweets containing “oral examination” or “oral health” were included in the results as well.

As pointed out by Yadav [1], there was inconsistency in the x-axis parameters (date) in Figure 5 in our published manuscript [2]. This was due to the difference in the starting point of the x-axis, which was the date when each piece of misinformation began to be disseminated on Weibo, while the ending point of the x-axis was March 16, 1 day before we carried out the search. Additionally, there was a typo in Table 2, in which “prosthetics” should be replaced by “prostheses.”

We have discussed the imbalance in dental service supply-demand during the epidemic and proposed that social media might be a potential solution at this unprecedented time. The tweets on Weibo reflected the great needs and short supply of dental treatment, but the specific fluctuation of dental demand-supply relationships with time was not able to be evaluated through Weibo. Nevertheless, social media can still serve as an important market indicator for both government policymakers and commercial companies to meet oral health needs during the pandemic.

Obviously, oral health information tweeted on social media is significantly influenced by the users’ socioeconomic status (educational background, job profile, income level) and oral health status (dental treatment experience, existing oral health problems). However, when performing social media–based studies, researchers have no access to this information due to the confidentiality and privacy of social media users, which means it is technically impossible to estimate the association between users’ background and the information they tweet about. This can be considered as an innate limitation of social media studies.

In China, Weibo is representative of all social media, especially during emerging epidemics when active users and tweets increase sharply [3,4]. According to the financial report of Sina Weibo for the first quarter of 2020 [5], the number of active users reached as high as 550 million per month and increased by 85 million compared with the same period last year. Therefore, we chose Weibo as the only social media platform for our study to avoid potential overlapping of users registered on other platforms. The large amount of Weibo users provided sufficient data for conducting the study.

The outbreak of COVID-19 in Wuhan, China, started on December 31, 2019. Since mid-March 2020, COVID-19 has been well controlled in Wuhan, with less than 10 new cases per day. Since then, dental hospitals and clinics have started to provide services to the public, leading to a significant reduction in tweets about COVID-19–related dentistry information. Accordingly, the scenario in Wuhan provides a unique and important social model to study the pandemic and related oral health information within a short time frame. Our study, covering the time period from December 31, 2019, to March 16, 2020, completely documents the changes in oral health information on social media from the beginning to the end of the outbreak in the world’s first COVID-19 epicenter.

The distribution of oral health information is closely related to the health care system of individual countries. We agree that future studies should be carried out to highlight further circulation of COVID-19–related oral information using a broader time frame and extended geographical locations. Comparisons of information between different geographical locations and different social media platforms in various languages will be of great value. It will not only provide insight into the global influence of the pandemic but will also shed light on the policy-making of health care systems around the world. This highlights a truly interesting and impactful research direction for the future.
